# Investigating miR-6880-5p in extracellular vesicle from plasma as a prognostic biomarker in endocrine therapy-treated castration-resistant prostate cancer

**DOI:** 10.1186/s12885-024-12460-x

**Published:** 2024-07-29

**Authors:** Jimin Lee, Jinhwa Hong, Ju Won Kim, Soonyoung Lim, Seung-Cheol Choi, Jeong-An Gim, Sung Gu Kang, Tae Il Noh, Kyong Hwa Park

**Affiliations:** 1 Division of Oncology/Hematology, Department of Internal Medicine, College of Medicine, Korea University Anam Hospital, 73, Goryeodae-Ro, Seongbuk-Gu, Seoul, 02841 Republic of Korea; 2 R&D Center for Companion Diagnostic, SOL Bio Corporation, Suite 510, 27, Seongsui-ro7-gil, Seongdong-gu, Seoul, 04780 Republic of Korea; 3 Medical Science Research Center, College of Medicine, Korea University Guro Hospital, Seoul, 08308 Republic of Korea; 4 Department of Urology, College of Medicine, Korea University Anam Hospital, 73, Goryeodae-Ro, Seongbuk-Gu, Seoul, 02841 Republic of Korea

**Keywords:** Biomarkers, Castration-Resistant, Extracellular Vesicles, MicroRNAs, Prostatic Neoplasmas

## Abstract

**Background:**

Advancements in the diagnosis, treatment, and surveillance of castration-resistant prostate cancer (CRPC) have progressed considerably, but a new biomarker that combines existing clinical and pathological data could be useful for a more precise diagnosis and prognosis. Some investigations have found that extracellular vesicle (EV)-derived miRNAs play crucial roles in various types of malignant tumors. The objective of this study was to explore EV miRNA and identify its biologic function as a biomarker for the diagnosis and prognosis of CRPC.

**Methods:**

Plasma samples were collected from five healthy donors (Control, CT) and 17 CRPC patients, categorizing into two groups based on their endocrine treatment response: partial response (PR; *n* = 10) and progressive disease (PD; *n* = 7). Candidate extracellular vesicle (EV) miRNAs were identified using miRNA microarray and RT-qPCR. The biological functions of the selected miRNAs were evaluated using the MTT assay, wound healing assay, trans-well assay, and RNA sequencing in CRPC cells after transient miRNA expression.

**Results:**

Microarray analysis revealed a significant downregulation of EV-miR-6880-5p in the PD samples compared to both CT and PR samples (*p* < 0.01). The expression of EV-miR-6880-5p in CRPC patients was decreased compared with that CT group (*p* = 0.0336) using RT-qPCR. In the PR group, EV-miR-6880-5p was increased at follow-up compared with the baseline (*p* = 0.2803), while in the PD group, it decreased at follow-up compared with the baseline samples (*p* = 0.4356). Furthermore, overexpression of miR-6880-5p hampered cell proliferation, migration, and invasion, downregulated pathways associated with tumor progression, and simultaneously upregulated pathways associated with cell growth and apoptosis in CRPC cells.

**Conclusions:**

EV-miR-6880-5p shows promise as a prognostic biomarker in patients with CRPC. Further, prospective validations are necessary to evaluate the potential of these candidate miRNAs.

**Supplementary Information:**

The online version contains supplementary material available at 10.1186/s12885-024-12460-x.

## Background

Prostate cancer (PCa) is the second most commonly occurring cancer in men and the fifth leading cause of cancer-related deaths worldwide. PCa is a heterogeneous tumor, and relatively little is known about its etiology [1]. Chemotherapy and androgen deprivation therapy are standard treatments for advanced prostate cancer and have provided considerable survival benefits. Despite the initial treatment, some patients progress to castration-resistant prostate cancer (CRPC). Patients with CRPC have an aggressive phenotype and a survival period of approximately three years [2]. For the definitive diagnosis of PCa, clinical stages and treatment selection are based on the prostate-specific antigen (PSA) level, tumor TNM (tumor, nodes, and metastases) stage and Gleason score of the prostate tissue shown through histopathological examination and transrectal ultrasound guided biopsy [3, 4]. However, PSA can also be detected in patients with non-malignant conditions, such as prostatitis, benign prostatic hyperplasia and prostate cysts, which can potentially result in unnecessary biopsy, overdiagnosis, and overtreatment [5]. Furthermore, the accuracy of tissue biopsies, which rely on random sampling templates, is significantly influenced by needle placement, leading to a higher likelihood of sampling errors [6].

The liquid biopsy includes analyzing the circulating macromolecules in body fluids, such as plasma, serum, saliva and urine. Compared with tissue biopsy, liquid biopsy is advantageous for being noninvasive and reduces risks such as pain, bleeding, and sepsis, which may occur during tissue biopsy. Liquid biopsy may use several types of biomarkers, such as PSA, cell-free DNA (cfDNA), circulating tumor cells (CTC), and extracellular vesicles (EVs) [7, 8]. CTC has been approved by the US Food and Drug Administration as prognostic biomarkers. However, CTC test is expensive and unfavorable for prostate cancer patients with ≥ 5 CTC per 7.5 mL blood [9]. EVs are more abundant and stable in the blood than CTC because they have a phospholipid bilayer structure compared with cfDNA [10]. EVs are small membranous vesicles (50–150 nm) formed by the endosomal system and released into the extracellular space. EV membranes express the EV markers CD9, CD63, CD81, ALIX, and TSG101 [11]. EVs also contain a variety of information about parent cells, including proteins, nucleic acids, and lipids [12]. EVs are involved in tumor progression, drug resistance, invasive and metastasis through the transport of tumor-related molecules to target cells and have been detected at high levels in fluid samples from various cancer cell types [13]. miRNA in EV have been reported to play an essential role in the tumor microenvironment by being associated with fibroblast proliferation, differentiation, migration, and angiogenesis [14, 15]. Recent studies have shown that EV-miRNA has the potential as a diagnostic, prognostic, and therapeutic biomarker with abnormal expression in urine and blood-based samples from patients with prostate cancer compared with that in healthy donors. However, its clinical role in CRPC development remains unclear [16, 17].

In this study, cancer-specific high-purity EVs and EV-miRNAs were isolated from the plasma of patients with CRCP according to their response to endocrine therapy. The roles and mechanisms of differentially expressed miRNAs were evaluated as potential biomarkers for the diagnosis and prognosis of plasma EV-miRNAs associated with CRPC.

## Methods

### Clinical samples

Blood samples were obtained from patients with prostate cancer (*n* = 17) diagnosed with CRPC, at Korea University Anam Hospital, Seoul, Republic of Korea, between 2016 and 2022 (Table 1). Responses to endocrine therapy (abiraterone or enzalutamide) were categorized as partial response (PR), stable disease, or progressive disease (PD) according to the Prostate Cancer Clinical Trials Working Group 3 criteria [18]. Paired samples of the baseline (pre-treatment) and at the time of response evaluation were collected and stored until analysis.

**Table 1:** Clinicopathologic characteristics of CRPC patients

		PR		PD	
		Baseline	Follow up	Baseline	Follow up
Total number		10		7	
age at the time of CRPC and specimen draw, median (range)		74.1 (67–89)	75 (68–90)	76.1 (65–83)	76.6 (66–83)
Treatment	Enzalutamide	6		3	
	Abiraterone	4		4	
PSA at diagnosis, ng/ml, median (range)		20.7 (1.6–41.9)	3.33 (0.031–10.21)	34.9 (1.39–151)	201.8 (2.79–1051)
Bone metastasis	Disseminated	4		5	
	Oligo	4		2	
	N	2		None	
Lymph node invasion	Y	3		4	
	N	7		3	
Gleason score at diagnosis	≤ 6	2		1	
	7	2		None	
	≥ 8	5		6	
	unknown	1		None	
TNM stage at diagnosis	IV	10		7	
Visveral metatstasis	Liver	None		1	
	Lung	None		None	
	Brain	None		None	

*CRPC* castration-resistant prostate cancer, *PD* progressive disease, *PR* partial response, *PSA* prostate-specific antigen, *TNM* tumor node metastasis

Samples obtained from healthy volunteers (*n* = 5) who had never been diagnosed with cancer were used as controls. All samples were collected and analyzed after obtaining written informed consent. Ethics approval for all procedures was obtained from the Korea University Anam Hospital (Approval no: 2016AN0287; October 24, 2016).

### Isolation of plasma from blood samples

Blood samples from patients with cancer and healthy donors were collected in tubes containing K2-EDTA, Na^+^-citrate barrier gel, or no additive. These tubes were centrifuged at 2,500 rpm for 7 min. The supernatant was then centrifuged at 16,000 rpm for 10 min at 4 ℃. Plasma was immediately stored in aliquots at –80 ℃ until analysis.

### Isolation of CD63^+^ extracellular vesicles from the plasma

Approximately 500 μL of plasma was subjected to three steps of pretreatment; first, samples were centrifuged at 1,200 × *g* for 20 min at 4 ℃. Then, the supernatant was re-centrifuged at 10,000 × *g* for 30 min at 4 ℃. Finally, the supernatant was filtered using a 0.2 μm syringe filter to remove large vesicles. EVs were isolated from pretreated samples using NeutraRelease kit according to the manufacturer’s protocol (SOL Bio, Inc., Seoul, Republic of Korea). Pretreated samples were mixed with the immunomagnetic beads immobilized with a calcium-binding protein conjugated with a capture antibody specific to CD63 [19] by a roller at RT for 2 h, eluted with elution buffer, and used immediately or stored at –80 ℃ until further use.

### Nanoparticle tracking analysis (NTA)

Nanoparticle tracking analysis (NTA) (NanoSight LM10, Malvern Panalytical, Malvern, UK) was used to confirm particle size distribution. EV samples were diluted 1:10 in PBS and loaded into a 1 mL syringe while avoiding bubble formation. The syringe was then loaded into the inlet port of the examination chamber. Subsequently, the laser was turned on, and the sample was captured by turning on the camera. The focus was adjusted according to the sample size. Three different frames were recorded, each consisting of 60 frames. The Batch Process option in the software was used to analyze the three different acquisitions.

### Transmission electron microscopy (TEM)

Transmission electron microscopy (TEM) was performed at the Korea Basic Science Institute (Chungcheongbuk-do, Republic of Korea). Plasma-derived EVs were layered onto a thin carbon foil-coated copper grid. The sample on the grid was contrasted with a UranyLess EM stain (Electron Microscopy Sciences, Pennsylvania, US) for 15 s. The grids were then washed with distilled water and dried. The sample images were acquired using a JEM-1400Plus electron microscope (JEOL, Tokyo, Japan).

### RNA extraction from EVs

RNA was isolated from EVs using the Total Exosome RNA and Protein Isolation Kit (Invitrogen, Carlsbad, CA, USA) per the manufacturer’s instructions with minor modifications. The extracted RNA was eluted with 30 μL of elution buffer. The quantity and quality of the resulting total RNA were measured using a nanodrop ND-1000 spectrophotometer (Thermo Fisher Scientific, Wilmington, DE, USA).

### RNA preparation and miRNA microarray assay

Total RNA, containing miRNAs, in EVs were isolated from the baseline plasma of patients with prostate cancer (PD: progressive disease; *n* = 5; PR: partial response; responder: *n *= 5) and sex-, age-, and race- matched healthy volunteers (control, CT; *n* = 5). The isolated total EV-RNAs were sent for miRNA microarray to Biocore (Seoul, Republic of Korea). RNA quality was assessed using the agilent 2100 BioAnalyzer (Agilent Technologies, Santa Clara, California, USA). No samples were excluded due to low RNA quality. The total RNA (10 ng) was labeled using the FlashTag Biotin RNA Labeling Kit (Affymetrix Inc., Santa Clara, CA, USA, Lot No.K19117HSR3) and hybridized to GeneChip miRNA 4.0 microarrays (Affymetrix Inc., Santa Clara, CA, USA) in the Affymetrix GeneChip Hybridization Oven according to the protocols provided by the manufacturer. Arrays were stained and washed in the Affymetrix GeneChip Fluidics Station 450 using the FS450_0002 fluidics protocol. All arrays were scanned with the Affymetrix GeneChip Scanner 3000 and raw analysis performed with Transcriptome Analysis Console™ (TAC) software to generate CEL files, which contained measure intensities for each probe on the array. The RMA-DABG algorithm was applied for data normalization. The Affymetrix miRNA 4.0 microarray contains 30,434 Total mature miRNA probe sets on the array, including 1,908 Mouse mature miRNA probe sets, while 2,578 are human (Data Sheet: GeneChip miRNA 4.0 and Affymetrix miRNA 4.1 Arrays.pdf). Affymetrix miRNA Arrays are designed to contain all miRNA in miRBase Release 20. The annotation file was obtained from the manufacturer’s web site. (https://www.thermofisher.com/order/catalog/product/902412).

### Microarray data analysis

Fifteen CEL files (ten experimental and five controls) were imported into the Gene Expression Workflow in GeneSpring GX version 14.9.1 (Agilent Technologies Inc.,). Background correction, log2 transformation, and probeset summarization were performed using default settings in GeneSpring software. Differential expression (DE) between PD group (experimental) and CT group (control) was predicted at the gene-level (probesets summarized into transcript clusters/genes). Also, Analysis of another group, i.e. PR group and CT group (control) is performed in same way. Unpaired t-test was respectively used to compare the individual gene expression data with respect to PD group versus control, and PR group versus control. DEGs were defined based on an absolute fold change equal to or greater than 1.3. A heatmap was generated using the R Studio software. ANOVA was performed for each group (CT, PD, and PR) among the 2578 genes. miRNAs (*n* = 12) were selected with an absolute fold change equal to or greater than 1.3. A *p* < 0.01 was considered a significant difference.

### Reverse transcription quantitative real-time polymerase chain reaction (RT-qPCR)

The complementary DNA synthesis was performed using the TaqMan MicroRNA Reverse Transcription kit (Applied Biosystems, Foster City, California, USA), and specific retro-transcription primers for miRNA were obtained from the TaqMan microRNA assay kit (Applied Biosystems). TaqMan RT-qPCR was conducted per the manufacturer’s instructions (Applied Biosystems). Each sample and miRNAs were analyzed in triplicates. The TaqMan assay used was has-miR-6880-5p (assay ID 467246_mat). The expression of miR-6880-5p was normalized to that of U6 small nuclear RNA.

### Cell culture

The normal prostate epithelial cell line RWPE-1 (RRID: CVCL_3791) was cultured in keratinocyte serum-free medium supplemented with 0.05 mg/mL bovine pituitary extract, 5 ng/mL epidermal growth factor and 1% penicillin–streptomycin. Human prostate cancer cell lines PC3 (RRID: CVCL_0035), DU145 (RRID: CVCL_0105), and LNCaP (RRID: CVCL_0395) cells were cultured in the RPMI-1640 medium supplemented with 10% fetal bovine serum and 1% penicillin–streptomycin. These cells were cultured at 37 °C in 5% CO_2_ under a humidified atmosphere. The cell lines were a generous gift from Prof. Won Jong Rhee (RWPE-1; Incheon National University, Incheon, Republic of Korea) and Prof. Seong-Gyu Ko (PC3, DU145, and LNCaP; Kyung Hee University, Seoul, Republic of Korea).

### miRNA isolation from cells

Total RNA, including small non-coding RNA, was extracted using the QIAzol reagent and the miRNeasy Mini kit (Qiagen, Hilden, Germany) according to the manufacturer’s protocol. A Nanodrop ND-1000 spectrophotometer (Thermo Fisher Scientific) was used to measure the concentration and purity of RNA obtained at an OD 260/280 ratio.

### Cell transfection

MiR-6880-5p mimic, miR-6880-5p inhibitor, and negative control were purchased from Ambion (Thermo Fisher Scientific). Cells were seeded in 6-well plates. After reaching 60–70% confluence, cells were transfected with either a mimic (10 nM) or inhibitor (50 nM) for each negative control and miR-6880-5p using Lipofectamine RNAiMAX according to the manufacturer’s protocol (Invitrogen). Transfected cells were incubated at 37 ℃ for 24 h before cells were lysed or sub-cultured.

### MTT assay

Briefly, the transfected cells (2.5 × 10^5^ cells/well) were seeded in 6-well plates and cultured for another 24, 48, and 72 h, and viable cell numbers were monitored using 3-(4,5 dimethylthiazol-2-yl)-2,5-disphenyltetrazolium bromide (MTT) staining. Absorbance was measured at 540 nm using a microplate reader (Bio-Rad, Hercules, CA, USA).

### Wound healing assay

The transfected cells (5 × 10^5^ cells/well) were seeded into 24-well plates and cultured overnight to assess the migration ability. The cell layer was scratched to create wounds using a sterile plastic pipette tip (200 μL) and washed in PBS. The cells were then cultured for 24 h. Wound images were acquired at different time points by optical microscopy.

### Trans-well assay

For invasion assays, we used a 24-well transwell chamber (8 μm; Corning, NY, USA). The transfected cells (1 × 10^5^ cells/well) were seeded in the upper chamber in 200 μL serum-free medium, whereas 750 μL of complete medium was added to the lower chamber. The two chambers were washed in PBS after 48 h, and the cells on the lower membrane surface were fixed with 4% paraformaldehyde and methanol and stained with 0.1% crystal violet. The upper membrane surface was removed using a cotton swab. Invasion images were acquired using an optical microscope and counted in four random fields.

### RNA isolation, library preparation, and sequencing

Total RNAs were isolated from DU145 cells transfected with the miR-6880-5p (1 nM, 6880_mi) and NC mimics using TRIzol reagent (Invitrogen; 10,296–010), according to the manufacturer’s protocol. Untreated DU145 cells were used as controls. The isolated total RNAs were sent for RNA-Sequencing analysis to EBIOGEN, Inc. (Seoul, Republic of Korea). RNA quality was assessed by an Agilent 2100 bioanalyzer using the RNA 6000 Nano Chip (Agilent Technologies, Amstelveen, The Netherlands), and RNA quantification was performed using ND-2000 Spectrophotometer (Thermo Inc., DE, USA). The library construction for those RNAs was carried out using the QuantSeq 3’ mRNA-Seq Library Prep Kit (Lexogen, Inc., Austria) in compliance with the instructions offered by manufacturer. In brief, each 500 ng total RNA were prepared and an oligo-dT primer containing an Illumina-compatible sequence at its 5’ end was hybridized to the RNA and reverse transcription was performed. After degradation of the RNA template, second strand synthesis was initiated by a random primer containing an Illumina-compatible linker sequence at its 5’ end. The double-stranded library was purified by using magnetic beads to remove all reaction components. The library was amplified to add the complete adapter sequences required for cluster generation. The finished library is purified from PCR components. High-throughput sequencing was performed as a single-end 75, ~ 10 M read using NextSeq 500 (Illumina, Inc., USA).

### Sequencing data analysis

The assessment of data quality was based on the phred quality scores across all bases (sanger/ Illumina 1.9 encoding). The quality of the generated sequences in FASTQ format was evaluated using the FASTQC (v0.11.8) program. The QuantSeq 3’ mRNA-Seq reads were aligned to the human reference genome (h19) using Bowtie2 (Langmead and Salzberg, 2012). Adaptor sequences and low-quality reads below Q20 were removed during trimming process. The align summary is shown in Supplementary Table 1. The alignment file was utilized for assembling transcripts, estimating their abundances, and identifying differential expression of genes. DEGs were determined based on counts from unique and multiple alignments using coverage in Bedtools (Quinlan AR, 2010). The RC (Read Count) data were processed based on TMM + CPM normalization method using EdgeR within R (R development Core Team, 2020) through Bioconductor (Gentleman et al.,2004). The ExDEGA software (EBIOGEN, Inc., Seoul, Republic of Korea) was applied to determine DEGs and gene ontology (GO) based on a 1.5-fold change and log2 > 4. Gene classification was performed based on database for annotation, visualization, and integrated discovery (DAVID; http://david.abcc.ncifcrf.gov/), Medline databases (http://www.ncbi.nlm.nih.gov/), kyoto encyclopedia of genes and genomes (KEGG) pathway (http://www.genome.jp/kegg/tool/map_pathway2.html), and search tool for the retrieval of interacting genes/proteins (STRING; http://www.string-db.org/). Heatmaps were analyzed using the multiexperiment viewer software (MeV, V4.9.0). Student’s t-test was utilized for determining DEGs. A *p* value less than 0.05 was deemed to indicate a significant difference.

### Statistical analysis

Data are presented as mean ± standard deviation (SD). A significance level of *p* < 0.05 was used to determine statistical significance. The statistically significant differences among the means were assessed by the two-tailed Student’s *t*-test or analysis of variance (ANOVA) followed by the Turkey test using GraphPad Prism software (version 8.0.2, La Jolla, CA, USA).

## Results

### Characterization of isolated extracellular vesicles (EVs) from plasma

**Fig. 1:**
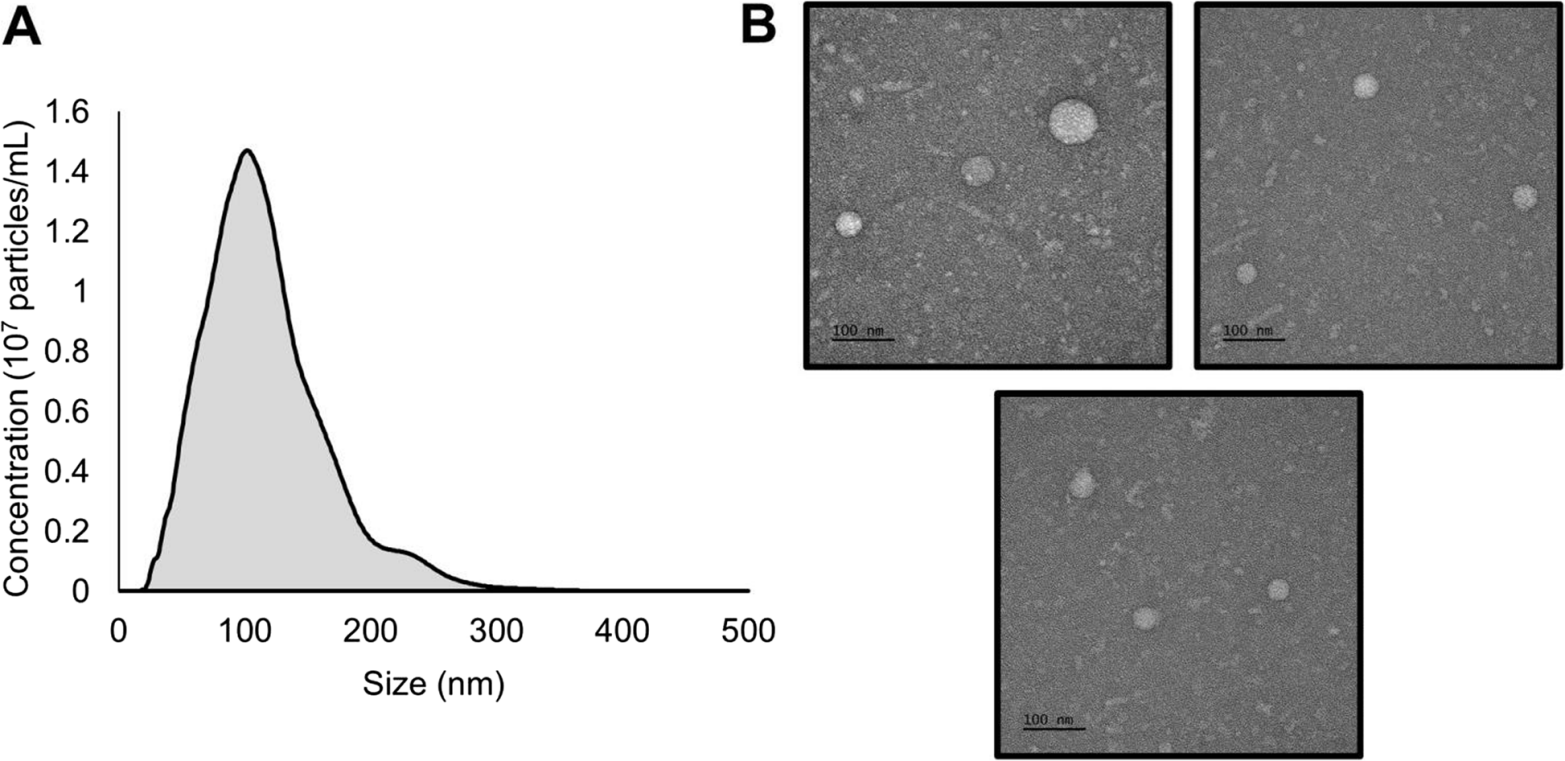
Characterization of extracellular vesicles (EVs) isolated from the plasma sample. Extracellular vesicles (EVs) were isolated from the plasma samples. **A** Nanoparticle tracking analysis (NTA) of 10 × diluted EVs from normal (noncancerous) plasma samples. **B** Transmission electron microscopy (TEM) image showing the phenotype of EVs isolated from plasma. The bars represent 100 nm

NTA and TEM were performed to confirm the characteristics of the EVs isolated from plasma. NTA shows that the average size of EVs separated from plasma samples was 117 ± 48 nm (mean ± SD) (Fig. 1A). Visualization of EV morphology using TEM revealed membrane-bound spherical structures with a uniform appearance (Fig. 1B).

Therefore, we confirmed that EVs isolated from plasma showed a circular structure (size: 50–150 nM), which is consistent with previously observed characteristics of extracellular vesicles.

### Discovery of differentially expressed miRNA in plasma EVs and castration-resistant prostate cancer cells

**Fig. 2:**
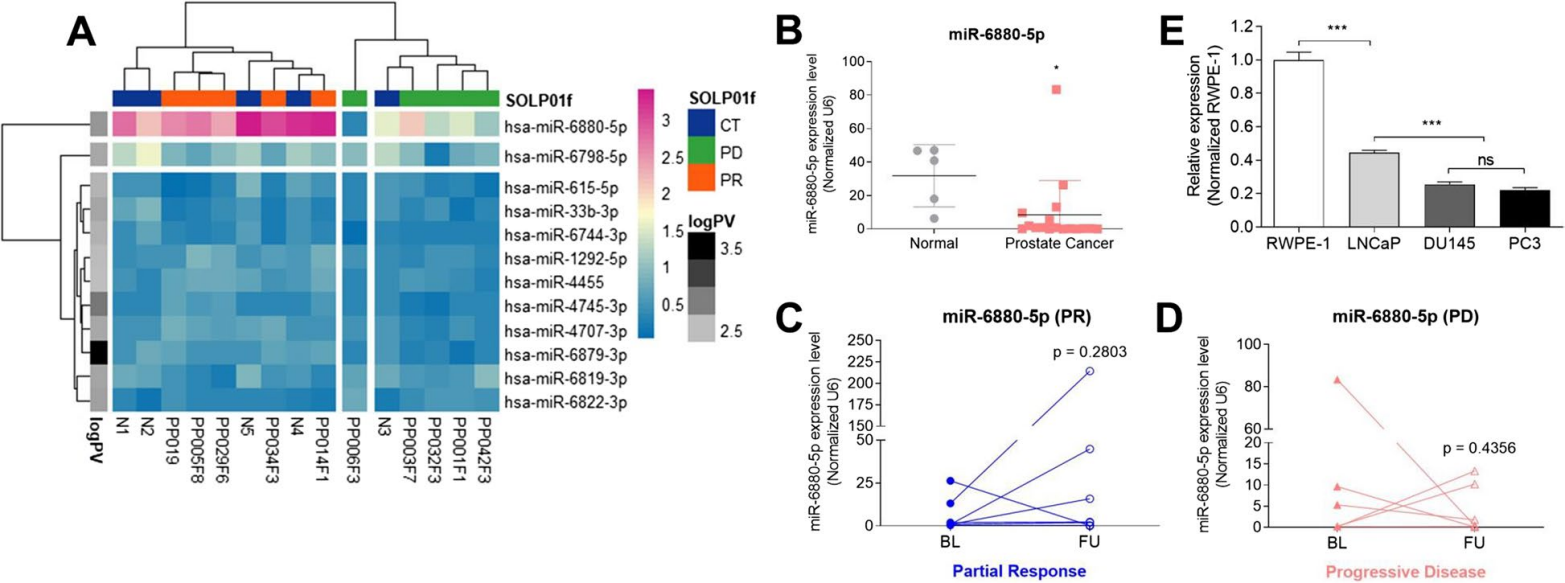
EV-miR-6880-5p expression is significantly lower in castration-resistant prostate cancer (CRPC). **A** A heatmap representing the differential miRNA expression in exosomes derived from the plasma of five healthy volunteers and 10 prostate cancer patients. Red indicates high relative expression, and blue indicates low relative expression. In the row annotation bar, the closer to the black color, the more statistically significant the difference in expression levels between the three groups. **B** The reliability of the EV-miRNA microarray was validated using RT-qPCR, assessing the average fold decrease of EV-miR-6880-5p in the 17 prostate cancer patients. **C**–**D** The graphs show the fold change of targeted EV-miRNAs grouped according to therapeutic responses in patients. **E** miR-6880-5p expression was detected using RT-qPCR from multiple prostate cell lines. A t-test was used to determine significance. *p < 0.05; **p < 0.01; ***p < 0.001

First, we used miRNA microarrays to identify candidate miRNAs in the EVs associated with prostate cancer. The patients were classified according to their clinical response to second- generation endocrine therapy for CRPC. Microarray data showed that 12 miRNAs from 2578 candidates were differentially expressed according to clinical response (*p* < 0.01; Fig. 2A). Among the 12 differentially expressed miRNAs, miR-6880-5p was significantly downregulated in patients in the PD group compared with the patients in the CT and PR groups (Fig. 2A). To validate the results from the miRNA microarray, RT-qPCR was performed to quantify miR-6880-5p expression in EVs from the plasma of healthy donors and patients with CRPC (Fig. 2B–D). The mean expression of EV-miR-6880-5p was significantly downregulated in patients with CRPC (mean ± SD; 8.42 ± 19.91 vs. 31.81 ± 16.61 in controls; *p* = 0.0336; Fig. 2B).

When the paired samples were analyzed according to therapeutic response, the expression level of EV-miR-6880-5p in the PR group was higher in the follow-up samples than in the baseline samples. However, the difference was not statistically significant (*p* = 0.2803, Fig. 2C). The expression levels of EV-miR-6880-5p in the PD group samples were downregulated in follow-up samples compared with the baseline samples; however, the difference was not statistically significant (*p* = 0.4356, Fig. 2D).

Next, we investigated whether there was differential expression of miR-6880-5p in prostate cancer cells with different degrees of malignancy and hormone sensitivity (RWPE-1: normal prostate epithelial cell line; LNCaP: hormone-naïve cell line; PC3 and DU134: CRPC cell lines) and observed that miR-6880-5p expression was significantly downregulated in prostate cancer cell lines compared with the normal prostate cell lines. The lowest expression of miR-6880-5p was observed in both CRPC cell lines (*p* < 0.001; Fig. 2E).

### Expression of miR-6880-5p significantly suppresses CRPC cell proliferation, migration, and invasion

**Fig. 3:**
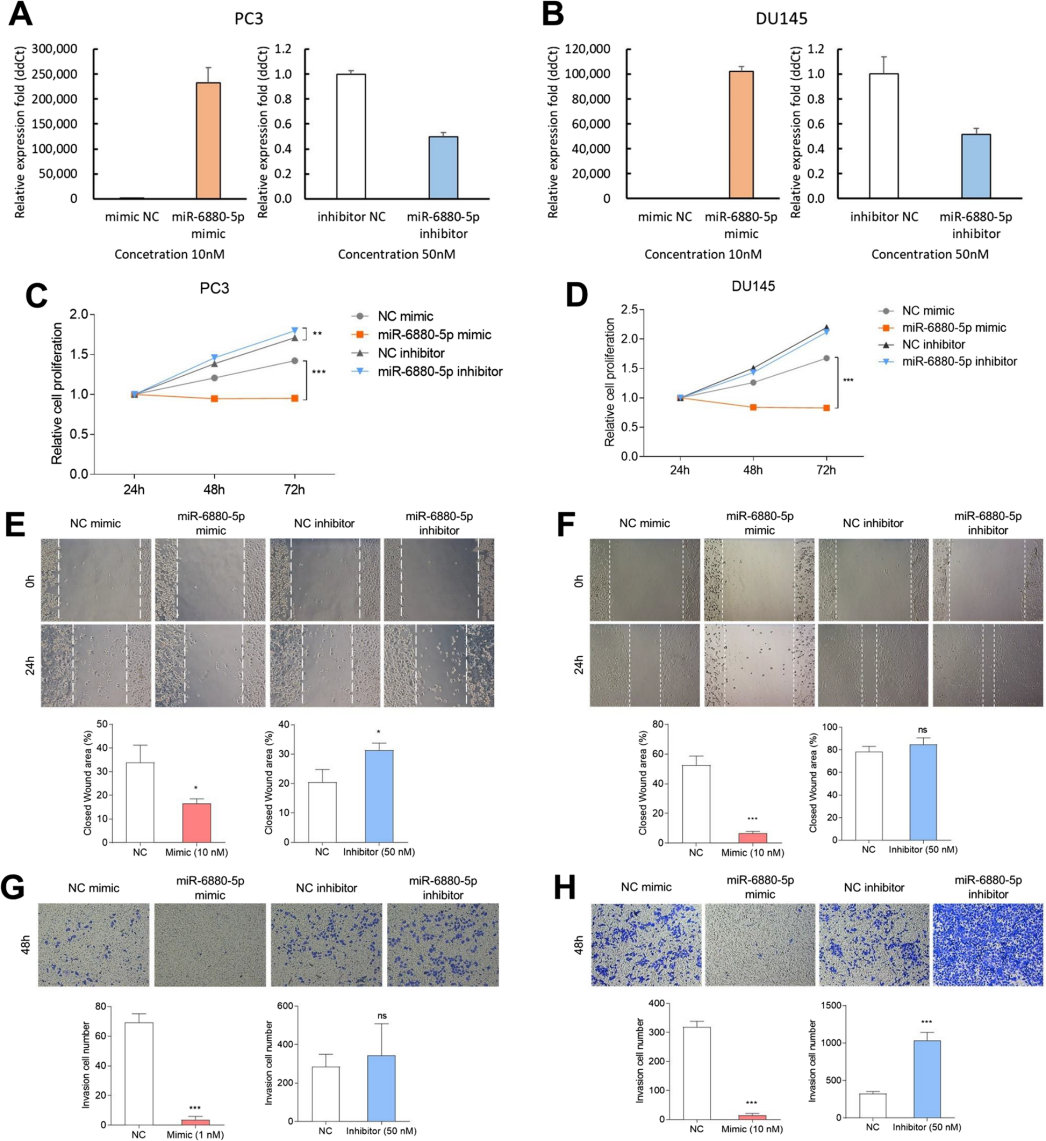
Transfection of a mimic or inhibitor of miR-6880-5p; each negative control was performed for cell function study in PC3 and DU145 cells. Expression of miR-6880-5p in (**A**) PC3 and (**B**) DU145 cells after transfection for 24 h with the mimic (10 nM) and the inhibitor (50 nM) compared with negative control. The effect of overexpression and inhibition of miR-6880-5p on cell proliferation in (**C**) PC3 and (**D**) DU145 cells was assessed using the MTT assay for 24, 48, and 72 h. Cell migration was assessed using a wound-healing assay. Images were captured at 0 and 24 h of incubation of transfected (**E**) PC3 and (**F**) DU145 cells. ImageJ was used to measure the percentage of closed wound areas. Cells were stained with crystal violet and counted manually in the transwell invasion assay. (**G**) PC3 cells. (H) DU145 cells *p < 0.05; **p < 0.01; ***p < 0.001 vs. negative control

The biological function of miR-6880-5p in CRPC cells (PC3 and DU145) was evaluated after transient miRNA expression (Fig. 3A, B).

The MTT assay showed that cell proliferation was significantly abrogated by the miR-6880-5p mimic in the PC3 and DU145 cell lines at 72 h compared with the NC mimic. The means of relative cell proliferation for the miR-6880-5p mimic compared with the NC mimic were 0.95 vs. 1.42 (PC3) and 0.83 vs. 1.67 (DU145) at 72 h, respectively (*p* < 0.001). However, the means of relative cell proliferation for the miR-6880-5p inhibitor compared with the NC inhibitor were 1.79 vs. 1.71 (PC3) and 2.11 vs. 2.2 at 72 h, respectively (Fig. 3C, D).

In the wound healing assay, compared with the NC mimic, the miR-6880-5p mimic showed a significant inhibitory effect on cell migration (mean ± SD: 16.5 ± 1.6% vs. 33.9 ± 6% in the NC mimic (PC3); *p* < 0.05; 6.7 ± 1% vs. 52.6 ± 5.3% in the NC mimic (DU145); *p* < 0.01). However, the miR-6880-5p inhibitor increased the closed wound area (mean ± SD: 31.4 ± 2% vs. 20.5 ± 3.5% in the NC inhibitor (PC3); *p* < 0.05; 84.7 ± 5.1% vs. 78.2 ± 4.2% in the NC inhibitor (DU145); ns) (Fig. 3E, F).

Furthermore, the results of the transwell assay showed that miR-6880-5p regulates the invasion of DU145 and PC3 cells through the overexpression or inhibition of their expression. The number of invasive cells remarkably increased in the miR-6880-5p inhibitor (mean ± SD: 343 ± 143 vs. 284 ± 57 cells in the NC inhibitor (PC3); *p* = ns; 1036 ± 92 vs. 325 ± 25 cells in the NC inhibitor (DU145); *p* < 0.001), whereas that in the mimic significantly abrogated (mean: 3 ± 2 vs. 69 ± 5 cells in the NC mimic (PC3); *p* < 0.001; 14 ± 6 vs. 318 ± 17 cells in the NC mimic (DU145); *p* < 0.001) (Fig. 3G, H).

Overall, miR-6880-5p overexpression suppressed cell proliferation, migration, and invasion, whereas the inhibition of miR-6880-5p promoted cell proliferation, migration, and invasion. These data indicated that miR-6880-5p has a tumor-suppressive function in CRPC.

### Comparison of DEGs through transcriptome analysis regulated by miR-6880-5p

**Fig. 4:**
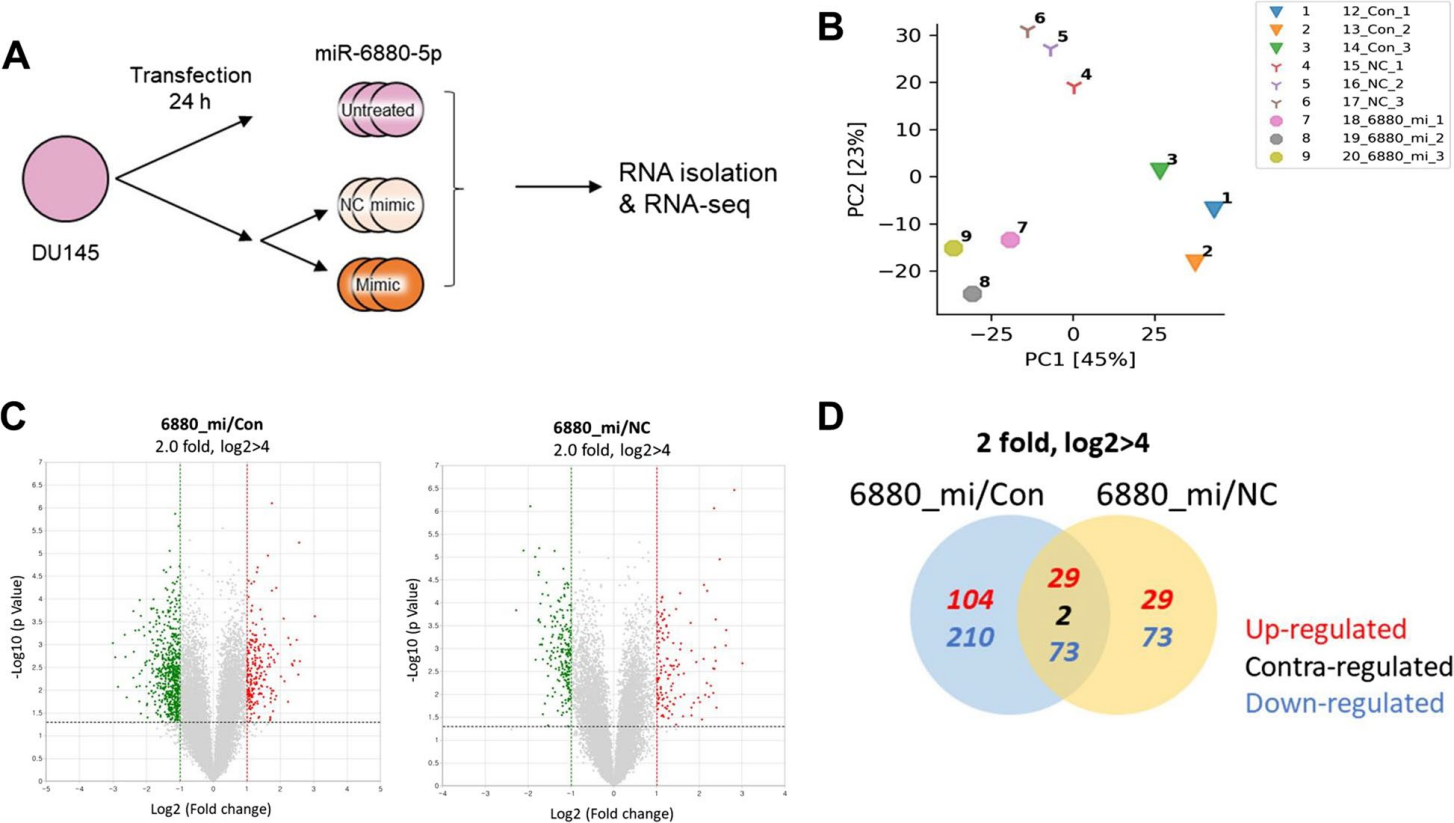
Analysis of differentially expressed genes (DEGs) from three libraries. **A** Schematic representation of transfection, RNA isolation, sequencing, and RNA-Seq analysis. Control (Con), untreated; negative control (NC); 6880_mi, transfection of the miR-6880-5p mimic. **B** Principal component analysis (PCA) results. miR-6880-5p mimics (6880_mi), Con, and NC show different distributions. **C** Total gene expression of 6880_mi compared with the Con (left) and NC (right) groups. **D** Venn diagram analysis between 6880_mi/Con (blue) and 6880_mi/NC (yellow)

Since the overexpression of miR-6880-5p abolished cellular functions, such as proliferation, migration, and invasion, global transcriptome changes due to the overexpression of miR-6880-5p were further investigated using transcriptome analysis (Fig. 4A).

Principal component analysis revealed a different gene expression distribution within each sample group (Fig. 4B). In addition, the expression of genes upregulated and downregulated by two-fold or more due to the miR-6880-5p mimic (6880_mi), shown in a Volcano plot, compared with the control and negative control (Fig. 4C). A total of 418 genes showed significant differential expression in the miR-6880-5p mimics compared with that in the control and 206 genes were significantly differentially expressed compared with the NC.

Overall, there were more downregulated genes (356 genes) among the differentially expressed genes than the upregulated genes (162 genes) (Fig. 4D).

### Pathway analysis shows the DEGs of miR-6880-5p in CRPC cells based on KEGG

**Fig. 5:**
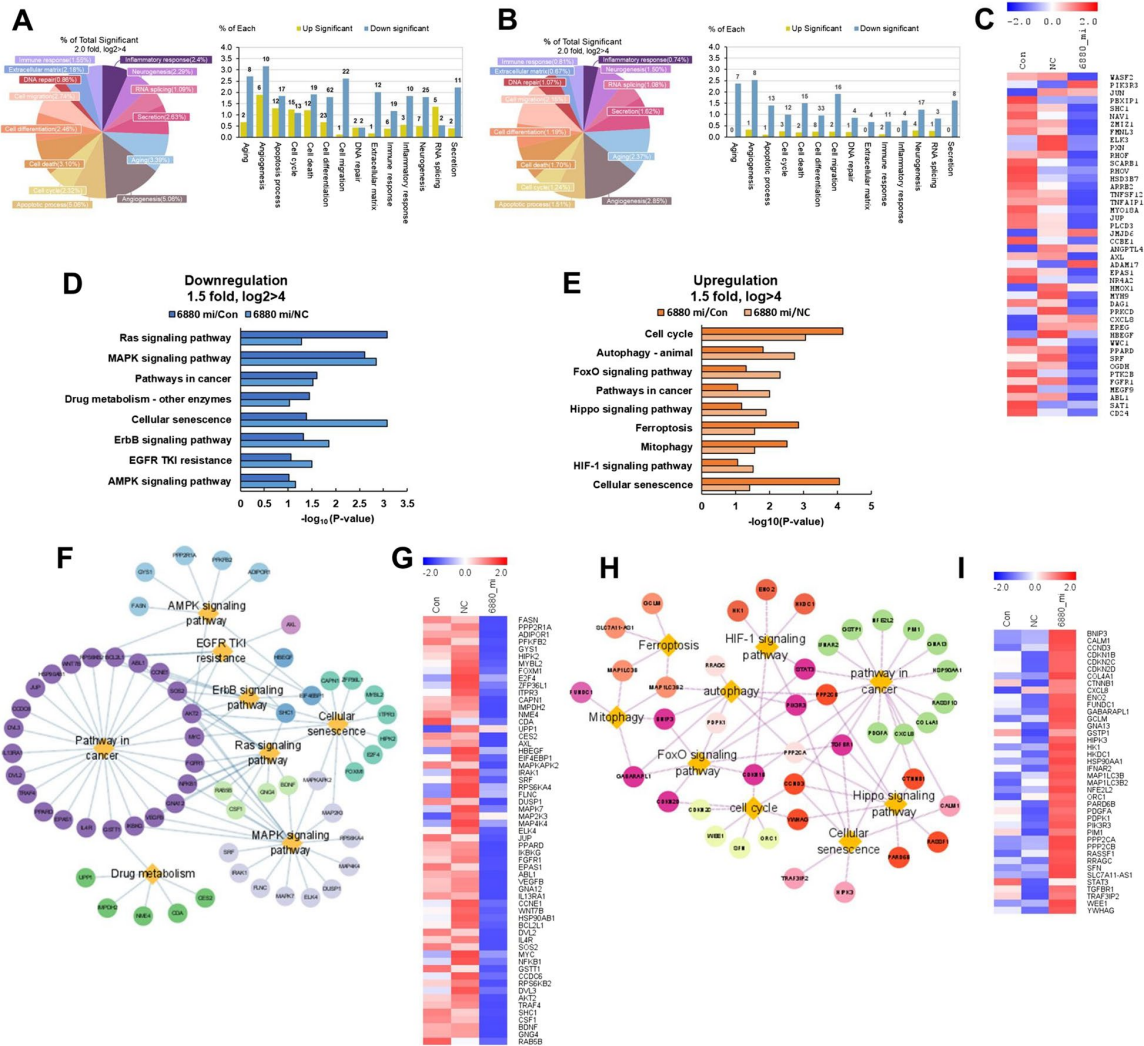
Identification pathway analysis of mRNA and miR-6880-5p interaction based on KEGG. The distribution of total significant DEGs in 14 GO categories in the transfected miR-6880 mimic (6880_mi) compared with the (**A**) control (con) and (**B**) negative control (NC) groups. **C** Heatmap analysis of downregulated genes associated with cell migration or angiogenesis compared with the control or NC. **D** Enriched KEGG pathways in the downregulated miR-6880-5p mimics compared with the control and NC. **E** Upregulated pathways. **F** GO result showing genes and pathways downregulated by miR-6880-5p mimics compared with the NC based on KEGG using STRING and Cytoscape. **G** Heatmap analysis of genes associated with downregulated pathways in miR-6880-5p mimics compared with the NC. **H** GO result showing genes and pathways upregulated by miR-6880-5p mimics compared with the NC based on KEGG using STRING and Cytoscape. **I** Heatmap analysis of genes associated with upregulated pathways in miR-6880-5p mimics compared with the NC

All significant DEGs were classified into 14 GO categories using the ExDEGA software. Most of these genes were downregulated to elucidate the functional association of cells with differentially expressed genes. In particular, pathways for angiogenesis (6880_mi/Con: 5.06%, 10 genes; 6880_mi/NC: 2.85%, 8 genes) and cell migration (6880_mi/Con: 2.74%, 22 genes; 6880_mi/NC: 2.15%, 16 genes) accounted for a high proportion, with a large number of downregulated genes compared with that in the control and NC groups (Fig. 5A, B). Further analysis revealed that the expression of genes associated with angiogenesis or cell migration was downregulated in the miR-6880-5p mimic group compared with that in the control and NC groups (Fig. 5C).

Moreover, we analyzed the transcriptomes using the KEGG pathway database to identify the signaling pathways of the DEGs associated with miR-6880-5p overexpression in detail. KEGG pathways primarily addressed overlapping pathways compared with the control and NC. The eight major pathways of DEGs in the downregulated genes were associated with the *Ras* signaling pathway, MAPK signaling pathway, pathway in cancer, drug metabolism, cellular senescence, ErbB signaling pathway, EGFR tyrosine kinase inhibitor (TKI) resistance, and AMPK signaling pathway (Fig. 5D). Conversely, the nine major pathways of the DEGs in the upregulated genes were associated with the cell cycle, autophagy, FoxO signaling pathway, pathway in cancer, Hippo signaling pathway, ferroptosis, mitophagy, HIF-1 signaling pathway, and cellular senescence (Fig. 5E). Network analysis revealed the interaction of eight major pathways through downregulated genes in the miR-6880-5p mimic group compared with the control and NC groups (Fig. 5F). Using the same method, we examined the interaction of the nine major pathways through the upregulated genes in the miR-6880-5p mimic group compared with the control and NC groups (Fig. 5H). Heatmap analysis revealed the expression of genes between the groups (Fig. 5G, I).

Overall, we found that the pathways downregulated by miR-6880-5p overexpression were closely related to tumor-promoting pathways, whereas the upregulated pathways were related to cell proliferation and apoptosis regulation. These results indicate that miR-6880-5p may function as a tumor suppressor.

### Clinical significance of miR-6880-5p among the patients with prostate cancer in the TCGA database

**Fig. 6:**
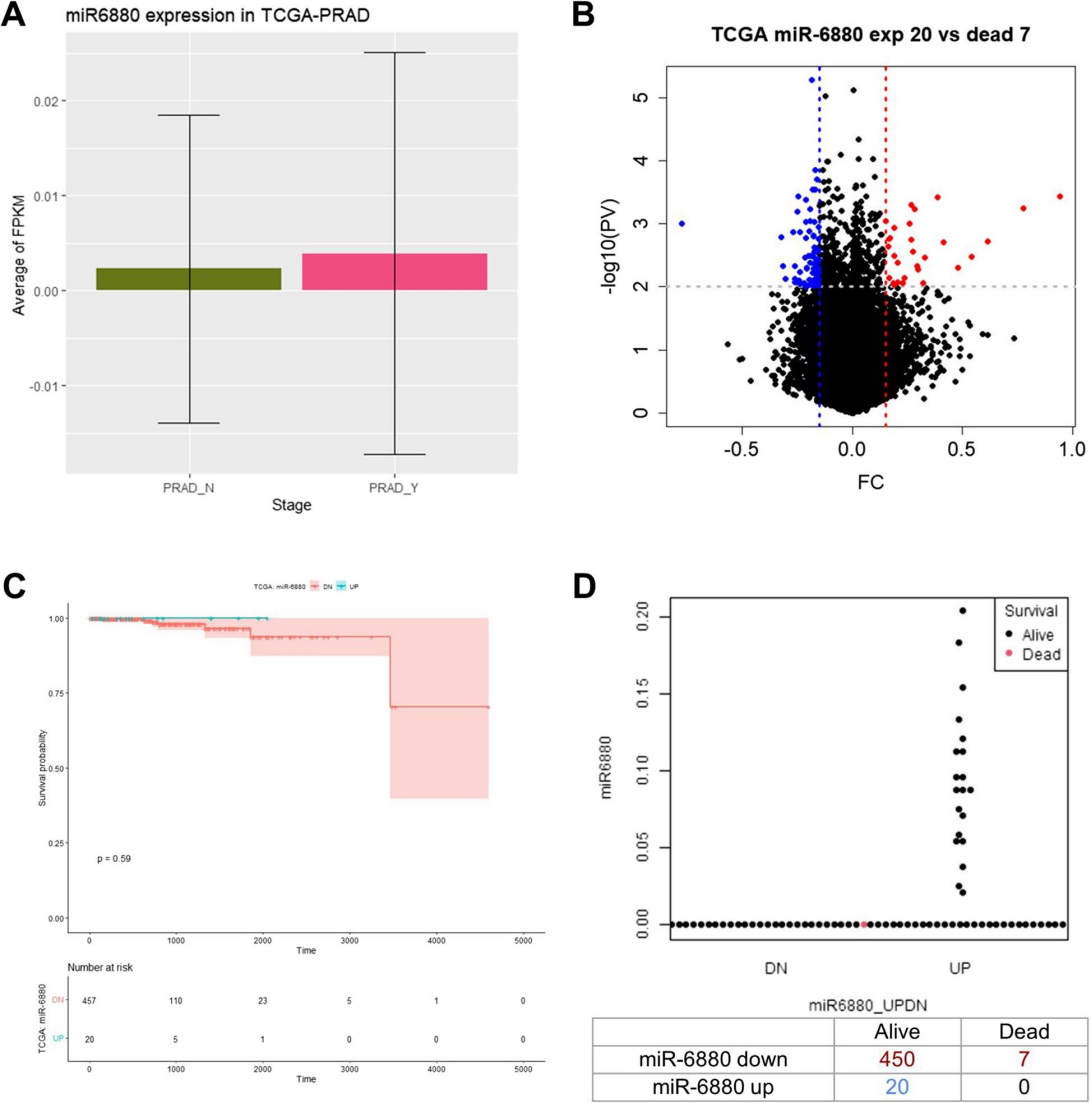
The Cancer Genomic Atlas Prostate Adenocarcinoma (TCGA-PRAD) database analysis of miR-6880-5p. **A** miR-6880-5p levels between normal tissue (PRAD_N) and prostate tissue (PRAD_Y). **B** A volcano plot showing the differential expression of miR-6880-5p in prostate cancer. **C** Kaplan–Meier analysis of overall survival based on tissue expressing miR-6880-5p in TCGA database. **D** Scatterplots of survival analysis by miR-6880-5p levels

We assessed whether the expression of miR-6880-5p in primary prostate cancer would have clinical significance using The Cancer Genome Atlas of Prostate Adenocarcinoma (TCGA-PRAD). The average miR-6880-5p (PRAD_Y) expression was higher in primary PC tissues than in normal tissues (PRAD_N) (Fig. 6A). Among the 477 samples, the expression of miR-6880-5p was undetectable in the majority (*n* = 457). However, 20 samples showed a high level of expression (Fig. 6B). Among the patients with low-miR-6880-5p-expressing tumors, 450 patients survived and 7 died. However, all 20 patients with high tumor expression levels survived (Fig. 6C, D). The difference in overall survival was not statistically significant in the log-rank test because of limited data in the TCGA dataset (*p* = 0.59).

## Discussion

Recent advance in treatment modalities for patients with CRPC have revolutionized treatment, and some treatment options have improved patient survivability [4, 20]. However, patients with CRPC show heterogeneous prognoses according to various clinical factors, including performance status, symptoms, and laboratory findings [18]. To accurately predict the diagnosis and prognosis of patients with CRPC, additional tests should be developed that integrate serum PSA levels with existing clinical pathological parameters.

In this study, EVs were isolated using CD63^+^ magnetic beads from plasma of patients with CRPC. miRNAs isolated from EVs were evaluated as potential prognostic biomarkers based on the clinical response of patients with CRPC. Among the candidate miRNAs, miR-6880-5p showed significantly lower expression in the plasma EVs of patients with CRPC than in healthy volunteers using RT-qPCR. Furthermore, in vitro functional studies using CRPC cell lines supported the potential biological role of miR-6880-5p. Overexpression of miR-6880-5p in CRPC cells downregulated pathways related to tumor promotion and upregulated pathways related to tumor suppression. Our results suggest that miR-6880-5p in EVs may serve as a sensitive miRNA suppressor for CRPC diagnosis.

Researchers have found that EVs in body fluids contain tumor characteristics, such as proteins, lipids, DNA, and RNA in various cancer types, suggesting vast opportunities for cancer diagnosis [12]. Many methods have been used to isolate EVs, including ultracentrifugation (UC), exosome precipitation, density gradients, and size-exclusion chromatography. However, these methods could be limited to exclusively isolating cancer-specific EVs due to the complex mixture of EVs and other components of the extracellular space [21]. In this study, we used a unique immune-isolation method developed by magnetic beads binding the calcium-binding protein conjugated with the capture antibody specific to CD63 after pretreatment using a 0.2 μm filter and UC. CD63, a surface marker of EVs, is upregulated in various carcinomas, including prostate cancer [22]. A previous study found that EVs isolated using this method can be used to diagnose early-stage malignant melanoma [19]. The isolated EVs were of high purity, adequate size, and morphology. EVs isolated using this method can be used to discover tumor-specific diagnostic and prognostic biomarkers in clinical samples.

miRNAs, a type of non-coding small RNA reported to have extensive biological effects, are known to be associated with tumor development [23]. For example, miR-665 is upregulated in EVs from the serum in hepatocellular carcinoma [14]. miR-21, widely studied in malignant tumors, is highly expressed and can promote cell proliferation and invasion in prostate cancer by inhibiting the expression of PTEN, a tumor suppressor gene [17]. In patients with CRPC, miR-423-3p, miR-1290, and miR-375 in plasma EVs have to be upregulated and associated with poor prognosis in previous studies [16, 24]. However, the mechanism underlying the effect of miRNAs on tumor biology in CRPC and their implementation mechanism as a biomarker in clinical practice remain unclear. In this study, we used microarray analysis to discover differentially expressed miRNAs in EVs isolated from the plasma of patients with CRPC compared to healthy volunteers. To validate the microarray results, RT-qPCR was conducted. The expression of miR-6880-5p in patients with CRPC was significantly lower than that in the CT group. Patients who did not respond to endocrine therapy (PD group) showed a lower expression of miR-6880-5p than those in the PR and healthy volunteer groups at baseline. With treatment, the expression of miR-6880-5p increased in patients showing a clinical response; however, statistical significance was not reached. The miR-6880-5p expression levels in the cell lines were also observed according to the order of endocrine sensitivity, in line with clinical observations; CRPC cell lines (DU145 and PC3 cells) exhibited the lowest miR-6880-5p expression compared with the hormone-naive (LNCaP cells) and normal (RWPE-1 cells) cell lines. These data suggest the potential to predict the grade of prostate cancer and the response to endocrine therapy based on miR-6880-5p expression.

miR-6880-5p decreased in the sera of patients with pancreatic and biliary tract cancers compared with the healthy controls [25]; however, its biological effect on cancer cells remains unclear. Herein, we showed that the overexpression of miR-6880-5p in CRPC cell lines significantly abrogated cell proliferation, migration, and invasion. Further analysis of the miRNA-mRNA regulatory network using miR-6880-5p-overexpressed DU145 cells was performed. Among the 14 GO categories, differentially expressed genes were significantly associated with angiogenesis and cell migration. Angiogenesis is associated with cell migration, invasion, and proliferation [26]. These results are consistent with that of our in vitro functional studies (Fig. 4). Functional annotation analysis revealed that downregulated pathways were associated with tumorigenesis and progression, whereas upregulated pathways were associated with tumor suppressor genes. In downregulated pathways, the Ras and MAPK signaling pathways have been reported to play pivotal roles in the development of metastatic prostate cancer by losing the function of PTEN and activating the PI3K pathway [27]. The ErbB signaling pathway has been reported to promote tumor growth by reactivating androgen receptor expression in patients with CRPC. It has been observed that lapatinib addition, an EGFR and ErbB2 inhibitor, significantly improved the response to abiraterone in CRPC [28]. The pathway upregulated by miR-6880-5p overexpression included pathways (FoxO signaling pathway, ferroptosis, autophagy, and mitophagy) associated with apoptosis and anti-tumor immunity [29, 30], although there are pathways that promote tumors. FoxO is a tumor suppressor gene associated with cell differentiation, apoptosis, cell cycle arrest, and DNA damage and repair [31]. Previous studies have shown that FoxO1 inhibits the androgen-independent activation of AR in prostate cancer [32]. Mitophagy acts as a tumor promoter or suppressor and largely depends on the status and subtype of the cancer cells [33]. However, our data showed that mitophagy is related to the pre-apoptotic pathway.

Our research has some limitations. This study is an exploratory pilot study rather than one based on statistical hypotheses. Therefore, the small sample size may not adequately reflect the influence of intervening factors. This means that the identified EV-miR-6880-5p needs to be validated in larger-scale clinical trials so that it can be used more clearly to diagnose CRPC patients and predict endocrine sensitivity. In addition, longer follow-up samples are required in future studies on response evaluation after treatment. Further, follow-up is needed to determine how the expression of our selected miRNAs changes with patient status after additional treatment. Lastly, to find out more about the prognostic value of CRPC, it is necessary to determine the expression levels of other miRNA markers or genetic parameters, in addition to miR-6880-5p.

## Conclusions

In conclusion, we found that miR-6880-5p in EVs isolated from the plasma acts as a suppressor miRNA in patients with CRPC. These novel findings suggest diagnostic and prognostic targets of liquid biopsies for patients with CRPC and provide novel insights into the underlying mechanisms of CRPC. However, further research is needed, including higher-quality investigations that follow standardized guidelines for the research design.

### Supplementary Information

Supplementary Material 1.

## Data Availability

No datasets were generated or analysed during the current study.

## Abbreviations

ANOVA: Analysis of variance
CRPC: Castration-resistant prostate cancer
cfDNA: Cell free DNA
CT: Control
CTC: Circulating tumor cells
DEG: Differentially expressed gene
DAVID: Database for annotation, visualization, and integrated discovery
EV: Extracellular vesicle
GO: Gene ontology
KEGG: Kyoto encyclopedia of genes and genomes
MTT: 3-(4,5 Dimethylthiazol-2-yl)-2,5-disphenyltetrazolium bromide
NC: Negative control
NTA: Nanoparticle tracking analysis
PC: Prostate cancer
PD: Progressive disease
PR: Partial response
PRAD: Prostate adenocarcinoma
PSA: Prostate-specific antigen
RT-qPCR: Reverse transcription quantitative real-time polymerase chain reaction
SD: Standard deviation
STRING: Search tool for the retrieval of interacting genes/proteins
TCGA: The cancer genome atlas
TEM: Transmission electron microscopy
TKI: Tyrosine kinase inhibitor
UC: Ultracentrifugation

## References

[1] Sung H, Ferlay J, Siegel RL, Laversanne M, Soerjomataram I, Jemal A, Bray F. Global Cancer Statistics 2020: GLOBOCAN Estimates of Incidence and Mortality Worldwide for 36 Cancers in 185 Countries. CA Cancer J Clin. 2021;71(3):209–49. 33538338 10.3322/caac.21660

[2] Chandrasekar T, Yang JC, Gao AC, Evans CP. Mechanisms of resistance in castration-resistant prostate cancer (CRPC). Transl Androl Urol. 2015;4(3):365–80. 26814148 10.3978/j.issn.2223-4683.2015.05.02 PMC4708226

[3] Bruinsma SM, Bangma CH, Carroll PR, Leapman MS, Rannikko A, Petrides N, Weerakoon M, Bokhorst LP, Roobol MJ. Movember GAP3 consortium: Active surveillance for prostate cancer: a narrative review of clinical guidelines. Nat Rev Urol. 2016;13(3):151–67. 26813955 10.1038/nrurol.2015.313

[4] Litwin MS, Tan HJ. The Diagnosis and Treatment of Prostate Cancer: A Review. JAMA. 2017;317(24):2532–42. 28655021 10.1001/jama.2017.7248

[5] Frånlund M, Arnsrud Godtman R, Carlsson SV, Lilja H, Månsson M, Stranne J, Hugosson J. Prostate cancer risk assessment in men with an initial P.S.A. below 3 ng/mL: results from the Göteborg randomized population-based prostate cancer screening trial. Scand J Urol. 2018;52(4):256–62. 30241447 10.1080/21681805.2018.1508166 PMC6298808

[6] Derin O, Fonseca L, Sanchez-Salas R, Roberts MJ. Infectious complications of prostate biopsy: winning battles but not war. World J Urol. 2020;38(11):2743–53. 32095882 10.1007/s00345-020-03112-3

[7] Ionescu F, Zhang J, Wang L. Clinical Applications of Liquid Biopsy in Prostate Cancer: From Screening to Predictive Biomarker. Cancers (Basel). 2022;14(7):1728. 35406500 10.3390/cancers14071728 PMC8996910

[8] Trujillo B, Wu A, Wetterskog D, Attard G. Blood-based liquid biopsies for prostate cancer: clinical opportunities and challenges. Br J Cancer. 2022;127(8):1394–402. 35715640 10.1038/s41416-022-01881-9 PMC9553885

[9] Cieślikowski WA, Antczak A, Nowicki M, Zabel M, Budna-Tukan J. Clinical Relevance of Circulating Tumor Cells in Prostate Cancer Management. Biomedicines. 2021;9(9):1179. 34572366 10.3390/biomedicines9091179 PMC8471111

[10] Pang B, Zhu Y, Ni J, Thompson J, Malouf D, Bucci J, Graham P, Li Y. Extracellular vesicles: the next generation of biomarkers for liquid biopsy-based prostate cancer diagnosis. Theranostics. 2020;10(5):2309–26. 32089744 10.7150/thno.39486 PMC7019149

[11] Campos-Silva C, Suárez H, Jara-Acevedo R, Linares-Espinós E, Martinez-Piñeiro L, Yáñez-Mó M, Valés-Gómez M. High sensitivity detection of extracellular vesicles immune-captured from urine by conventional flow cytometry. Sci Rep. 2019;9(1):2042. 30765839 10.1038/s41598-019-38516-8 PMC6376115

[12] Fujita Y, Yoshioka Y, Ochiya T. Extracellular vesicle transfer of cancer pathogenic components. Cancer Sci. 2016;107(4):385–90. 26797692 10.1111/cas.12896 PMC4832849

[13] Becker A, Thakur BK, Weiss JM, Kim HS, Peinado H, Lyden D. Extracellular Vesicles in Cancer: Cell-to-Cell Mediators of Metastasis. Cancer Cell. 2016;30(6):836–48. 27960084 10.1016/j.ccell.2016.10.009 PMC5157696

[14] Qu Z, Wu J, Wu J, Ji A, Qiang G, Jiang Y, Jiang C, Ding Y. Exosomal miR-665 as a novel minimally invasive biomarker for hepatocellular carcinoma diagnosis and prognosis. Oncotarget. 2017;8(46):80666–78. 29113334 10.18632/oncotarget.20881 PMC5655229

[15] Wang J, Ni J, Beretov J, Thompson J, Graham P, Li Y. Exosomal microRNAs as liquid biopsy biomarkers in prostate cancer. Crit Rev Oncol Hematol. 2020;145: 102860. 31874447 10.1016/j.critrevonc.2019.102860

[16] Huang X, Yuan T, Liang M, Du M, Xia S, Dittmar R, Wang D, See W, Costello BA, Quevedo F, et al. Exosomal miR-1290 and miR-375 as prognostic markers in castration-resistant prostate cancer. Eur Urol. 2015;67(1):33–41. 25129854 10.1016/j.eururo.2014.07.035 PMC4252606

[17] Yang Y, Guo JX, Shao ZQ. miR-21 targets and inhibits tumor suppressor gene PTEN to promote prostate cancer cell proliferation and invasion: An experimental study. Asian Pac J Trop Med. 2017;10(1):87–91. 28107872 10.1016/j.apjtm.2016.09.011

[18] Scher HI, Morris MJ, Stadler WM, Higano C, Basch E, Fizazi K, Antonarakis ES, Beer TM, Carducci MA, Chi KN, et al. Trial Design and Objectives for Castration-Resistant Prostate Cancer: Updated Recommendations From the Prostate Cancer Clinical Trials Working Group 3. J Clin Oncol. 2016;34(12):1402–18. 26903579 10.1200/JCO.2015.64.2702 PMC4872347

[19] Choi DY, Park JN, Paek SH, Choi SC, Paek SH. Detecting early-stage malignant melanoma using a calcium switch-enriched exosome subpopulation containing tumor markers as a sample. Biosens Bioelectron. 2022;198: 113828. 34847362 10.1016/j.bios.2021.113828

[20] Nuhn P, De Bono JS, Fizazi K, Freedland SJ, Grilli M, Kantoff PW, Sonpavde G, Sternberg CN, Yegnasubramanian S, Antonarakis ES. Update on Systemic Prostate Cancer Therapies: Management of Metastatic Castration-resistant Prostate Cancer in the Era of Precision Oncology. Eur Urol. 2019;75(1):88–99. 29673712 10.1016/j.eururo.2018.03.028

[21] Doyle LM, Wang MZ. Overview of Extracellular Vesicles, Their Origin, Composition, Purpose, and Methods for Exosome Isolation and Analysis. Cells. 2019;8(7):727. 31311206 10.3390/cells8070727 PMC6678302

[22] Folkmanis K, Junk E, Merdane E, Folkmane I, Folkmanis V, Ivanovs I, Eglitis J, Jakubovskis M, Laabs S, Isajevs S, et al. Clinicopathological Significance of Exosomal Proteins CD9 and CD63 and DNA Mismatch Repair Proteins in Prostate Adenocarcinoma and Benign Hyperplasia. Diagnostics (Basel). 2022;12(2):287. 35204378 10.3390/diagnostics12020287 PMC8871402

[23] Wang Q, Xu K, Tong Y, Dai X, Xu T, He D, Ying J. el miRNA markers for the diagnosis and prognosis of endometrial cancer. J Cell Mol Med. 2020;24(8):4533–46. 32150330 10.1111/jcmm.15111 PMC7176884

[24] Guo T, Wang Y, Jia J, Mao X, Stankiewicz E, Scandura G, Burke E, Xu L, Marzec J, Davies CR, et al. The Identification of Plasma Exosomal miR-423-3p as a Potential Predictive Biomarker for Prostate Cancer Castration-Resistance Development by Plasma Exosomal miRNA Sequencing. Front Cell Dev Biol. 2020;8: 602493. 33490068 10.3389/fcell.2020.602493 PMC7817948

[25] Kojima M, Sudo H, Kawauchi J, Takizawa S, Kondou S, Nobumasa H, Ochiai A. MicroRNA markers for the diagnosis of pancreatic and biliary-tract cancers. PLoS ONE. 2015;10(2): e0118220. 25706130 10.1371/journal.pone.0118220 PMC4338196

[26] Melegh Z, Oltean S. Targeting Angiogenesis in Prostate Cancer. Int J Mol Sci. 2019;20(11):2676. 31151317 10.3390/ijms20112676 PMC6600172

[27] Mulholland DJ, Kobayashi N, Ruscetti M, Zhi A, Tran LM, Huang J, Gleave M, Wu H. Pten loss and RAS/MAPK activation cooperate to promote EMT and metastasis initiated from prostate cancer stem/progenitor cells. Cancer Res. 2012;72(7):1878–89. 22350410 10.1158/0008-5472.CAN-11-3132 PMC3319847

[28] Gao S, Ye H, Gerrin S, Wang H, Sharma A, Chen S, Patnaik A, Sowalsky AG, Voznesensky O, Han W, et al. ErbB2 Signaling Increases Androgen Receptor Expression in Abiraterone-Resistant Prostate Cancer. Clin Cancer Res. 2016;22(14):3672–82. 26936914 10.1158/1078-0432.CCR-15-2309 PMC4947432

[29] Ashrafizadeh M, Paskeh MDA, Mirzaei S, Gholami MH, Zarrabi A, Hashemi F, Hushmandi K, Hashemi M, Nabavi N, Crea F, et al. Targeting autophagy in prostate cancer: preclinical and clinical evidence for therapeutic response. J Exp Clin Cancer Res. 2022;41(1):105. 35317831 10.1186/s13046-022-02293-6 PMC8939209

[30] Wang Y, Ma Y, Jiang K. The role of ferroptosis in prostate cancer: a novel therapeutic strategy. Prostate Cancer Prostatic Dis. 2023;26(1):25–9. 36056183 10.1038/s41391-022-00583-w PMC10023567

[31] Shan Z, Li Y, Yu S, Wu J, Zhang C, Ma Y, Zhuang G, Wang J, Gao Z, Liu D. CTCF regulates the FoxO signaling pathway to affect the progression of prostate cancer. J Cell Mol Med. 2019;23(5):3130–9. 30873749 10.1111/jcmm.14138 PMC6484331

[32] Gheghiani L, Shang S, Fu Z. Targeting the PLK1-FOXO1 pathway as a novel therapeutic approach for treating advanced prostate cancer. Sci Rep. 2020;10(1):12327. 32704044 10.1038/s41598-020-69338-8 PMC7378169

[33] Guan Y, Wang Y, Li B, Shen K, Li Q, Ni Y, Huang L. Mitophagy in carcinogenesis, drug resistance and anticancer therapeutics. Cancer Cell Int. 2021;21(1):350. 34225732 10.1186/s12935-021-02065-w PMC8256582

