# Supplementary material for: Investigating miR-6880-5p in extracellular vesicle from plasma as a prognostic biomarker in endocrine therapy-treated castration-resistant prostate cancer
Source: BMC Cancer. 2024 Jul 29;24:909. doi: 10.1186/s12885-024-12460-x (PMC11285227; doi:10.1186/s12885-024-12460-x)
Supplement: Supplementary file 1 — Supplementary Material 1. [file 12885_2024_12460_MOESM1_ESM.docx]

| Sample | Processed_read | Mapped_read | Mapping_rate |
| --- | --- | --- | --- |
| Con_1 | 18318691 | 17206232 | 93.93% |
| 6880_mi_2 | 18403219 | 17332127 | 94.18% |
| NC_3 | 20225413 | 19070116 | 94.29% |
| Con_3 | 20721872 | 19574923 | 94.47% |
| 6880_mi_1 | 18716004 | 17696009 | 94.55% |
| Con_2 | 19104022 | 18077226 | 94.63% |
| NC_2 | 18096324 | 17137214 | 94.70% |
| NC_1 | 20349777 | 19320425 | 94.94% |
| 6880_mi_3 | 19905528 | 18943529 | 95.17% |

**Supplementary Table. 1** Align summary
